# Cerebral Amyloid Angiopathy-Related Inflammation in the Immunosuppressed: A Case Report

**DOI:** 10.3389/fneur.2019.01283

**Published:** 2019-12-06

**Authors:** Thomas Nelson, Bo Leung, Serguei Bannykh, Kevin S. Shah, Jignesh Patel, Oana M. Dumitrascu

**Affiliations:** ^1^Department of Neurology, Cedars-Sinai Medical Center, Los Angeles, CA, United States; ^2^Division of Neuropathology, Department of Pathology, Cedars-Sinai Medical Center, Los Angeles, CA, United States; ^3^Division of Heart Transplantation, Department of Cardiology, Cedars-Sinai Medical Center, Los Angeles, CA, United States

**Keywords:** cerebral amyloid angiopathy-related inflammation, mycobacteria, immunosuppression, sarcoidosis, transplantation

## Abstract

Cerebral amyloid angiopathy-related inflammation (CAA-ri) is an immune-mediated disorder of the central nervous system characterized by an inflammatory response to amyloid-beta (Aβ) deposition within cerebral blood vessel walls. Immunosuppressive therapy is the mainstay of treatment. We present a case of CAA-ri in a subject already on immunosuppressive therapy after orthotopic heart transplantation (OHT). A 57-year-old man 8 months post-OHT for sarcoid cardiomyopathy developed headaches and staring spells while hospitalized for disseminated mycobacterial infection. His brain MRI revealed bi-hemispheric T2-weighted fluid-attenuated inversion recovery white matter hyperintensities and widespread microhemorrhages. Two weeks later, he developed gait ataxia and alterations in mental status, and repeat brain MRI showed more extensive confluent white matter hyperintensities. Leptomeningeal and cortex biopsy revealed changes consistent with amyloid angiitis, with perivascular and intramural histiocyte and lymphocyte collections. Mass spectroscopy confirmed Aβ deposition. Notably, the patient was on immunosuppression with daily 5 mg oral prednisone and tacrolimus before biopsy. After high-dose intravenous followed by oral corticosteroids, he demonstrated significant clinical and radiographic improvement. No relapse was noted despite the relatively rapid tapering of the prednisone therapy over 3 months, as mandated by his systemic infection. Despite the lack of a standard treatment protocol for CAA-ri, case series have reinforced the benefit of prolonged courses of glucocorticoids as single agent or in combination with other immunomodulatory agents. Hence, management of CAA-ri in patients with disseminated mycobacterial infections or OHT is challenging. Our case is unique, as review of existing literature has not revealed any similar cases of patients on chronic immunosuppression at the time of CAA-ri diagnosis, which one would expect to protect against this disorder. In addition, CAA-ri in association with cardiopulmonary sarcoidosis was not previously reported; however, a common immunopathogenic mechanism may exist.

## Background

Cerebral amyloid angiopathy-related inflammation (CAA-ri) is a rare disorder of the central nervous system (CNS) that arises from development of autoantibodies to amyloid-beta protein (Aβ) within the walls of the leptomeningeal and cortical blood vessels (1–5). It affects immunocompetent individuals who present with non-specific symptoms or focal neurological deficits, which typically elicits a broad etiological investigation until the condition is recognized (6–8). The presumptive diagnosis is based on clinical and neuroimaging data, whereas the definitive diagnosis is histological (9). The mainstay of CAA-ri management is with prolonged courses of immunosuppression, although no standard treatment protocol has been established to date. We present the case of a patient who developed CAA-ri despite chronic immunosuppression for sarcoidosis and orthotopic heart transplantation (OHT).

## Case Presentation

**Figure 1:**
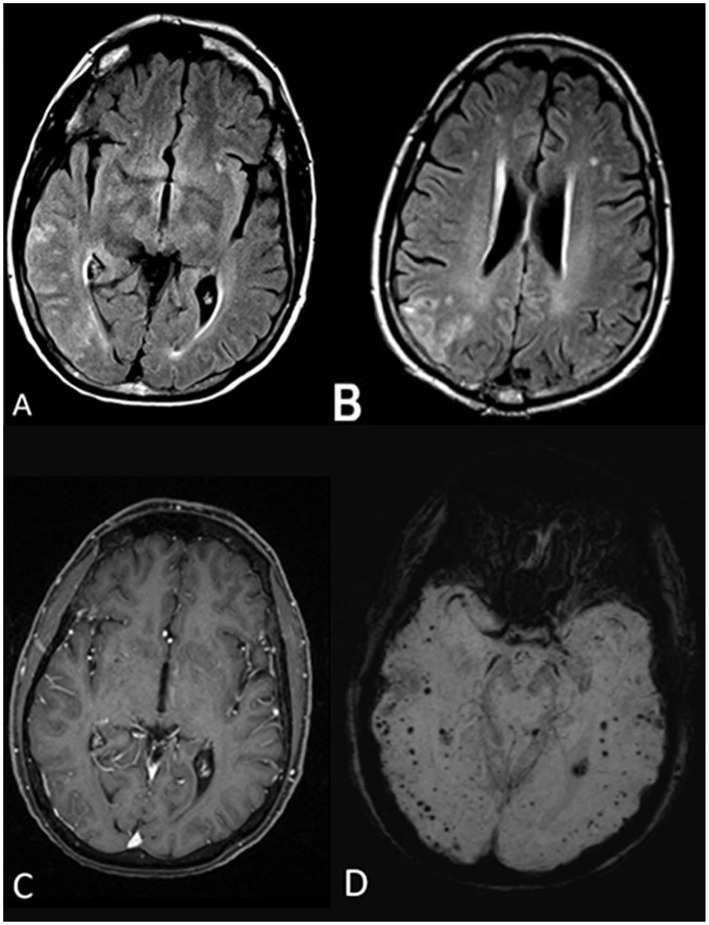
Initial MRI brain without and with gadolinium. Axial fluid-attenuated inversion recovery (FLAIR) sequences **(A,B)** illustrate scattered T2 FLAIR hyperintensities in the right temporal and parietal lobes involving the cortex and subcortical white matter. The lesions did not demonstrate contrast enhancement, and no leptomeningeal enhancement was noted either **(C)**. Susceptibility-weighted imaging (SWI) sequence shows numerous bihemispheric microhemorrhages, in the cortical and subcortical white matter **(D)**.

**Figure 2:**
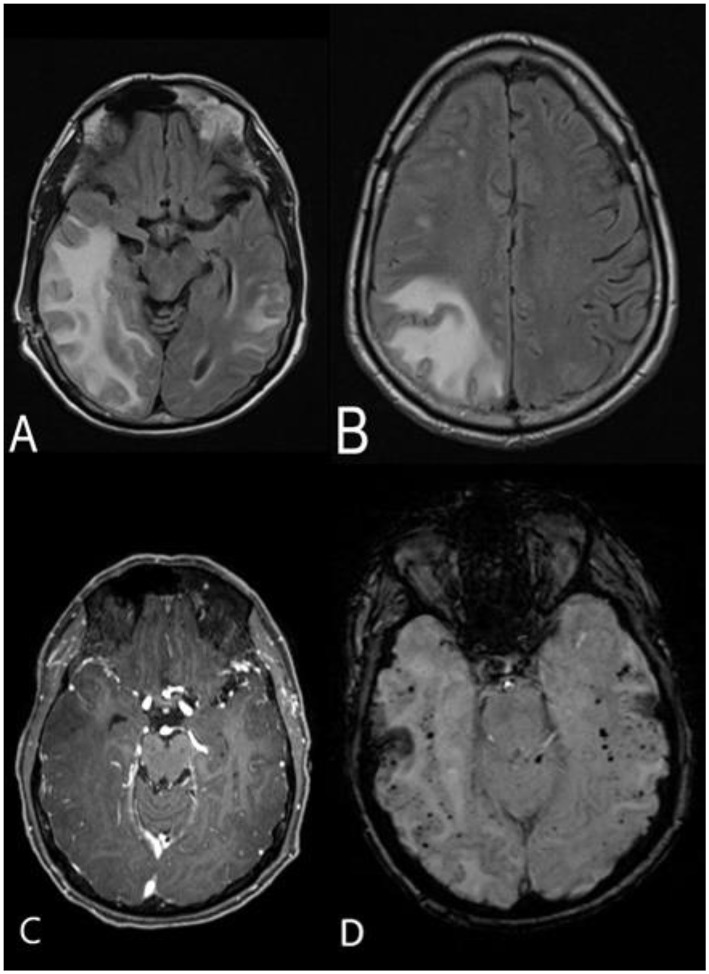
MRI brain without and with contrast approximately 3 weeks after the initial scan. Axial FLAIR sequence demonstrates significant progression of the hyperintense lesions in the right temporal, occipital, and fronto-parietal areas, with mild mass effect on the right lateral ventricle and midline shift **(A,B)**. No contrast enhancement is appreciated **(C)**. SWI sequence shows stable, diffuse, bihemispheric microhemorrhages **(D)**.

**Figure 3:**
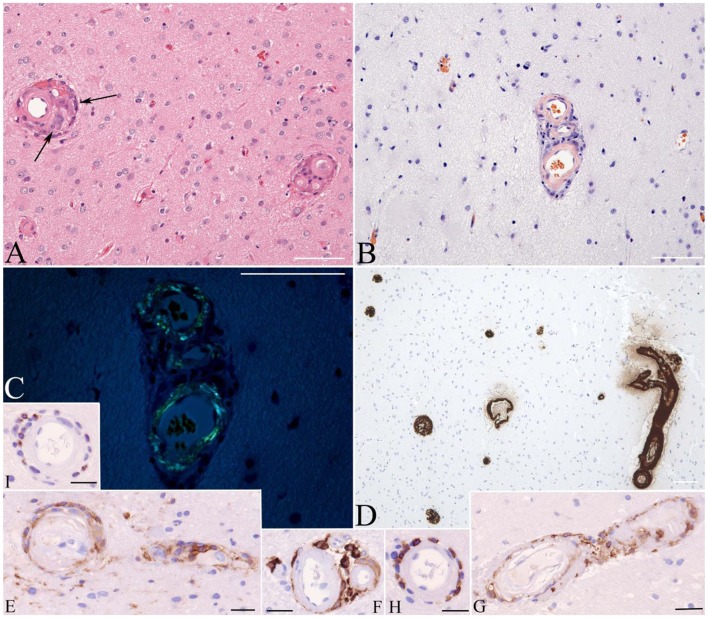
Brain biopsy stained with hematoxylin and eosin discloses thickened microvasculature with amorphous hyaline-like material replacing the media and associated with mononuclear as well as granulomatous infiltrates (arrows on **A**). Congo Red stain without **(B)** and with polarization **(C)** highlights congophilic **(B)** bi-refringent **(C)** amyloid deposits, immunoreactive with antibody to Aβ **(D)**. Immunophenotyping of the inflammatory cells shows staining for macrophage marker CD163 **(E–G)**, T lymphocyte marker CD8 **(H)**, and cytotoxic granule marker T-cell intracellular antigen (TIA) **(I)**. Bars: 100 μ.

**Figure 4:**
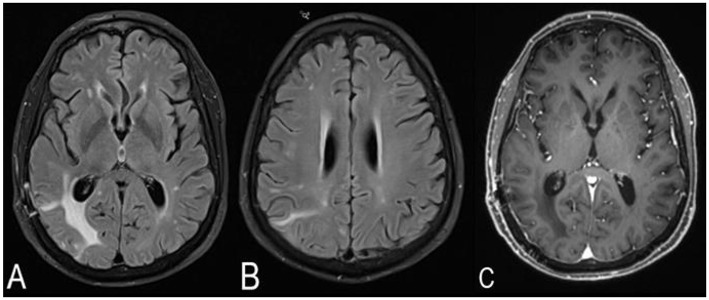
MRI brain without and with contrast 1 month after the brain biopsy and treatment with high-dose corticosteroids. The right cerebral T2-weighted fluid-attenuated inversion recovery (T2/FLAIR) hyperintensities have decreased in size, and the midline shift has resolved **(A,B)**. No contrast enhancement is noted **(C)**.

A 57-year-old male geologist with history of cardiopulmonary sarcoidosis complicated by dilated cardiomyopathy presented approximately 8 months after OHT, with a 6-weeks history of diffuse painful, raised skin lesions and open sores of the hands. His immunosuppressive regimen consisted of a combination of methotrexate, leflunomide, tacrolimus, and 5 mg prednisone daily. Blood and skin cultures isolated *Mycobacterium hemophilum*. The patient was treated with intravenous antibiotics for 11 days when he developed complex partial seizures, headaches, blurry vision, and mood changes. Brain MRI with contrast demonstrated abnormal T2-weighted fluid-attenuated inversion recovery signal hyperintensity in the cortical and subcortical right temporal, parietal, and occipital lobes without contrast enhancement (Figure 1). Susceptibility-weighted imaging sequences revealed innumerable chronic microhemorrhages in the bilateral infra- and supratentorial cerebral hemispheres. Early considerations included neurosarcoidosis, CNS infection, and lymphoma. However, cerebrospinal fluid studies were non-specific with two white blood cells, two red blood cells, glucose of 70, and mildly elevated protein of 47. Angiotensin-converting enzyme, meningoencephalitis PCR panel (including *Escherichia coli* K1, *Haemophilus influenza, Listeria monocytogenes, Neisseria meningitidis*, Enterovirus, Herpes simplex virus 1 and 2, human herpesvirus 6, varicella zoster virus, parechovirus, and *Cryptococcus neoformans*/*Cryptococcus gatii*), Coccidioides and *Cryptococcus* antibodies, mycobacteria, viral and fungal cultures, flow cytometry, and cytology testing of the cerebrospinal fluid were unrevealing. Owing to disseminated mycobacterial infection, his immunosuppressive regimen was narrowed to tacrolimus (at goal level, 4–8 ng/ml) and prednisone 5 mg daily. Two weeks after discharge, the patient returned with progressively worsening gait ataxia. Repeat neuroimaging was significant for more extensive, confluent, non-enhancing right hemispheric white matter hyperintensities; and stable diffuse microhemorrhages (Figure 2). Spinal MR imaging was unremarkable. Right parietal leptomeningeal and cortex biopsy revealed changes consistent with CAA-ri. The vasculature showed presence of congophilic polarizable birefringent deposits, which stained strongly with anti-Aβ 6F/3D monoclonal antibody, and intramural and perivascular collections of histiocytes and lymphocytes (Figure 3). Tissue acid-fast and Gram stains were negative. Liquid chromatography–mass spectroscopy confirmed the presence of Aβ/A4 peptide deposition. Despite systemic mycobacterial infection, the pathological diagnosis of CAA-ri prompted immediate treatment with intravenous methylprednisolone 1 g daily for 5 days followed by 60 mg oral prednisone. Steady clinical improvement followed shortly, and significant radiographic improvement was noted on repeat neuroimaging a month later (Figure 4). Subsequently, the decision was made to taper oral prednisone over 3 months, as mandated by the underlying systemic infection. No neurological recurrence was noted to date, at 6-months follow-up.

## Discussion

Our case is unique in presenting the development of pathologically proven CAA-ri in a patient with sarcoidosis and on chronic immunosuppression in a post-cardiac transplantation setting.

Cerebral amyloid angiopathy (CAA) is localized amyloidosis affecting cerebral blood vessels through Aβ deposition. A small subset of CAA patients develops a secondary vasculitis due to autoantibody formation to Aβ, leading to a syndrome known as CAA-ri (1, 3, 4). Although histology provides a definitive diagnosis, large case series have posited that clinical features and typical MRI findings may obviate the need for invasive testing and can stratify patients into probable or possible CAA-ri (7, 9). Our patient's clinicoradiographical presentation was non-specific, with potential for complications due to his chronic immunosuppression (such as CNS infection, malignancy, posterior reversible encephalopathy syndrome), or post-OHT status (cerebral microhemorrhages). Hence, given rapid progression of the white matter lesions and clinical deterioration, a histological diagnosis was obtained. The complex presentation not only delayed the diagnosis but also posed a therapeutic challenge. No standard treatment regimens have been established to date for CAA-ri, but prolonged courses of glucocorticoids as single-agent therapy or in conjunction with other immunosuppressants have been proposed (2, 6, 7, 10). There is clinical equipoise about the selection of an appropriate agent, dose, and duration of treatment. However, CAA-ri tends to require longer courses of immunosuppression—usually 6 months—with rare recurrence. Given our patient's systemic mycobacterial infection, the decision was made for a relatively shortened, 3-months course of oral prednisone taper. The robust and sustained clinicoradiographic response observed despite relatively fast corticosteroid tapering may suggest that this approach could be considered in other similar, complex patients.

To our knowledge, there are no other similar reports of patients on chronic immunosuppression at the time of diagnosis of CAA-ri, which one would expect to protect against this disorder. There is no evidence that our patient was on suboptimal immunosuppression considering his opportunistic disseminated mycobacterial infection and absence of cardiac allograft rejection.

In addition, review of existing literature did not reveal any prior cases of patients with both sarcoidosis and CAA-ri. However, there is evidence of comorbid autoimmune disease with sarcoidosis and possible potentiation of inflammatory responses seen with CAA-ri. Specifically, prior series have associated sarcoidosis with autoimmune thyroid disease, Sjogren disease, ankylosing spondylitis, systemic sclerosis, giant cell arteritis, and inflammatory myopathy, among other disorders (11–14). A large case series reported CAA-ri patients with comorbid Grave disease, pernicious anemia, rheumatoid arthritis, and autoimmune hepatitis (7). It is unclear whether there is a direct or indirect interaction between these conditions, but it is likely that a genetic predisposition for a robust and disproportionate inflammatory response may precipitate this autoimmune damage. Such a genetic predisposition has been proposed between APOE ε4/ε4 genotype and increased Aβ fibril deposition, particularly perivascularly, that may potentiate the inflammatory response seen in CAA-ri (2, 13, 15, 16). Moreover, there appears to be a contribution of amyloid, specifically serum amyloid A, to granuloma formation in sarcoidosis (17). Serum amyloid A may act to trap the etiologic antigen, leading to chronic sarcoidosis, and highlights a possible pathogenic similarity in both sarcoidosis and CAA-ri development (17, 18).

## Conclusion

Our report illustrates that CAA-ri may occur despite seemingly adequate immunosuppression in patients with an underlying predisposition for autoimmune disease. Faster corticosteroid tapering may be considered in complex CAA-ri patients with systemic infections, with close clinical and radiological surveillance.

## Data Availability Statement

The datasets generated for this study are available on request to the corresponding author.

## Ethics Statement

Written informed consent was obtained from the individual(s) for the publication of any potentially identifiable images or data included in this article.

## Author Contributions

TN, BL, and OD contributed conception and design of the study. BL and SB organized the figures. TN wrote the first draft of the manuscript. TN, BL, SB, KS, JP, and OD wrote sections of the manuscript. OD critically revised the final manuscript draft. All authors contributed to manuscript revision, read and approved the submitted version.

### Conflict of Interest

The authors declare that the research was conducted in the absence of any commercial or financial relationships that could be construed as a potential conflict of interest.
